# Efficacy of nitazoxanide to treat natural *Giardia* infections in dogs

**DOI:** 10.1186/s13071-017-1998-7

**Published:** 2017-01-31

**Authors:** Mario Moron-Soto, Lilia Gutierrez, Héctor Sumano, Graciela Tapia, Yazmin Alcala-Canto

**Affiliations:** 1 Departamento de Parasitología, Facultad de Medicina Veterinaria y Zootecnia, Universidad Nacional Autónoma de México, Av. Universidad 3000, Delegación Coyoacán, Ciudad de México, C.P. 04510 Mexico; 2 Departamento de Fisiología y Farmacología, Facultad de Medicina Veterinaria y Zootecnia, Universidad Nacional Autónoma de México, Av. Universidad 3000, Delegación Coyoacán, Ciudad de México, C.P. 04510 Mexico; 3 Departamento de Genética y Bioestadística, Facultad de Medicina Veterinaria y Zootecnia, Universidad Nacional Autónoma de México, Av. Universidad 3000, Delegación Coyoacán, Ciudad de México, C.P. 04510 Mexico

**Keywords:** *Giardia*, Nitazoxanide, Dogs, Parasite, Efficacy

## Abstract

**Background:**

*Giardia* parasites cause gastrointestinal disease in humans, dogs, and many other animals worldwide. The treatment of dogs for giardiasis requires further investigation to ascertain levels of drug efficacy and the possibility of adverse side effects. Nitazoxanide (NTZ) has shown good clinical anti-*Giardia* activity in humans, yet it has not been evaluated for the treatment of giardiasis in dogs.

**Methods:**

Thirty-five dogs, naturally infected with *Giardia* were divided into five groups (*n* = 7): dogs in group NTZ1, NTZ2, and NTZ3 were treated with a single oral dose of 37.5 mg/kg, 75 mg/kg, and 150 mg/kg, respectively, of NTZ on days 0 and 14. The fourth group was treated with a commercially available regimen that includes a combination of pyrantel, praziquantel, and febantel (FEB) administered orally for three consecutive days. Additionally, an untreated control group was established. *Giardia* cysts from the stool of each dog were quantified on days -3, 0, 5, 7, 9, 11, 14, 18, 25, and 28. Biochemical parameters were evaluated in all dogs, before the first treatment and after concluding the experiment.

**Results:**

Shedding of *Giardia* cysts was reduced in all treated groups when compared to untreated controls (*P* < 0.01). However, NTZ2, NTZ3, and FEB had a lower risk during the study. Furthermore, NTZ was also effective against another protozoan, *Cryptosporidium* spp. at doses of 75 mg/kg and 150 mg/kg, in contrast to the combination of febantel + pyrantel + praziquantel. Biochemical parameters of treated animals, namely, aspartate transaminase and alanine transaminase enzymes, remained within physiological ranges.

**Conclusions:**

Based on these results, the implementation of NTZ as a treatment for giardiasis in dogs is proposed. The administration of a single dose is an important advantage of NTZ because it reduces workload, particularly in animals placed in shelters and kennels, where handling of large numbers of animals is required, and personnel is frequently scarce.

## Background

Canine giardiasis is caused by isolates of *Giardia* from four of the eight recognized genetic assemblages [1]. Of these, assemblage A and B have zoonotic potential [2]. *Giardia* has been described as the most commonly found parasite in dogs [3], though many infections are subclinical and remain undetected. Cysts and trophozoites of *Giardia* are shed in the faeces of all infected hosts [4], contaminating food, water, and the environment [5, 6]. Once a cyst has been ingested by a susceptible host, two trophozoites are released within the small intestine that will either remain free in the intestinal lumen or adhere to enterocytes causing damage to the microvilli and inducing the clinical signs of the infection. Trophozoites differentiate into cysts as they near the colon, completing their life-cycle in approximately 8 days [7]. Giardiasis is associated with a wide range of clinical signs, from diarrhea and malabsorption to a mild gastrointestinal discomfort [8]. However, it has been stated that giardiasis should be treated in all positive dogs so that they do not remain a source of infection for other animals and humans [2].

Although not approved by many regulatory agencies, such as Food and Drug Administration (FDA), metronidazole is one of the most commonly used drugs to treat giardiasis in dogs [9]. The use of tinidazole, a derivative of metronidazole, has also been reported [10], yet it is unavailable in many countries. Adverse drug effects are common with these agents and include nausea, vomiting, diarrhea, general discomfort, and loss of appetite [11]. Fenbendazole and febantel possess activity against *Giardia* [10]. Albendazole has also been used to control this parasite, but bone marrow suppression has been associated with the use of this benzimidazole derivative in dogs and cats [12, 13]. There are other drugs with giardicidal activity, such as furazolidone, which can cause hemolysis in individuals with a deficiency in the glucose 6-phosphate dehydrogenase enzyme. Quinacrine is another available giardicidal compound, though it is poorly tolerated because it causes nausea, vomiting, and cramps [14]. Paramomycin is another potential giardicidal compound, but there is limited information on its use [15–18]. While metronidazole has been regarded as the drug of choice for treating giardiasis, resistance has been documented in human cases of *Giardia* infection, possibly due to mechanisms that allow the protozoan to tolerate the oxidative stress caused by this drug [9].

Nitazoxanide (NTZ) is a relatively new drug that was approved by FDA in 2014 for the treatment of giardiasis in humans. It is also effective against *Trichomonas* spp., *Entamoeba histolytica*, *Clostridium difficile*, *Clostridium perfringens*, *Helicobacter pylori*, and *Campylobacter jejuni* [19]. It has been shown that NTZ has a similar efficacy for eliminating *Giardia* when compared to metronidazole, but has fewer adverse effects [20–23]. To the best of our knowledge, there are no formal studies on the effect of NTZ against clinical infections of *Giardia* in dogs. Therefore, the current trial was carried out to assess its efficacy against this protozoan, using febantel as FDA approved standard.

## Methods

### Nitazoxanide preparation

Nitazoxanide (assigned purity, 99.53%) was kindly supplied by Shin Yang–Hangzhou, Shinyang Samwoo Fine Chemical CO. (Ningbo, China). Differential scanning calorimetric (DSC), infrared absorption spectroscopy, and ^1^H and ^13^C nuclear magnetic resonance spectroscopy revealed a lack of impurities. A 30% suspension was made using sodium benzoate, sucrose, xanthan gum, microcrystalline cellulose and carboxymethylcellulose sodium, anhydrous citric acid, sodium citrate dihydrate, acacia gum, sugar syrup, FD&C Red #40 and Artificial Powdered Meat Flavor PC-0170 (PF Inc., Florida, USA).

### Animals

The work was carried out adhering to the guidelines of the Institutional Committee for Use and Care of Experimental Animals of the institution, according to the Mexican Official Regulation NOM-062-ZOO-1999 [24]. Sixty-five dogs, selected from a shelter in Texcoco, State of Mexico, Mexico, were screened for the presence of *Giardia* by microscopic examination of faeces after zinc sulfate centrifugation before the commencement of the study. Initially, 40 animals were included as they tested positive to *Giardia* on three occasions before the trial. Dogs infected with pathogens other than gastrointestinal parasites, as well as those with other ailments were excluded from the study. Dogs shedding fewer than 10 *Giardia* cysts per examined slide were excluded from the trial. Thus, 14 male and 21 female dogs, 1.42 ± 0.5 years old on average and weighing 14.4 ± 2.5 kg (average) were included in this study. All dogs had lived in the shelter for more than 3 months and had not received any antiparasitic treatment in at least 3 months. Upon their arrival to the shelter, they were treated with a single dose of oxibendazole and niclosamide paste (Vitaminthe® Reforzado, Virbac, Mexico). Animals were allocated to individual rooms (1.5 × 3.0 m), separated from each other with wire mesh and with 70% humidity. They were fed commercial food without nutraceuticals (Ol’ Roy Adulto, México) *ad libitum,* had free access to water and soft bedding. The enclosures were cleaned daily and the dogs’ comfort was ensured.

### Experimental design

A randomized non-inferiority study was conducted for the treatment of canine giardiasis, comparing the efficacy of three different doses of nitazoxanide administered every 14 days, to that of a FDA-approved reference antiparasitic product containing pyrantel embonate, febantel and praziquantel (Drontal Plus®, Bayer Animal Health, Mexico) administered for three consecutive days according to the manufacturer’s recommended dosage. The giardicidal efficacy of the investigational product was required to be equal to the reference product to achieve non-inferiority.

This trial was conducted according to the guidelines of the World Association for the Advancement of Veterinary Parasitology (WAAVP) for dose determination studies. This document states that one internationally accepted design includes a minimum of three groups receiving different dosages of the investigational drug together with an untreated control group [25]. Nitazoxanide has been experimentally administered to *Giardia*-infected dogs previously at an oral dose of 25 mg/kg twice a day, with no efficacy, as 37.5 to 50% of dogs that completed the experimental protocol, remained positive for *Giardia* [26]. Considering that 50 mg/kg/day was not effective against giardiasis in dogs, a dose of 75 mg/kg was selected, hoping to achieve a greater efficacy. Furthermore, previous experiments tested the anthelmintic efficacy of NTZ orally administered to dogs at a dose of 75–200 mg/kg, and a high efficacy against cestodes and nematodes was obtained [27].

All dogs were weighed on an electronic scale before treatment and blood samples were collected for hematological and biochemical analysis before drug dosing and 2 days after the last treatment. For allocation purposes, systematic random sampling was used. Animals were ranked from lowest to highest *Giardia* cyst count determined on day zero (0) before treatments and assigned to one of the five groups (*n* = 7).

Groups 1 (NTZ1), 2 (NTZ2), and 3 (NTZ3) were treated orally with the nitazoxanide paste preparation at a dose of 37.5 mg/kg, 75 mg/kg, or 150 mg/kg, respectively, on days 0 and 14 of the trial. The FEB group received the FDA-approved reference commercial antiparasitic product formulated as a tablet, which included a dose of 15 mg/kg of febantel, 5 mg/kg of praziquantel, and 14.4 mg/kg of pyrantel embonate (Drontal Plus®, Bayer Animal Health, Mexico) on days 0, 1, and 2 of the experiment, as recommended by the manufacturer. An untreated control group (CTRL) was also established, and received a placebo paste that contained the following excipients: sodium benzoate, sucrose, xanthan gum, microcrystalline cellulose and carboxymethylcellulose sodium, anhydrous citric acid, sodium citrate dihydrate, acacia gum, sugar syrup, FD&C Red #40 and artificial powdered meat flavor PC-0170. All dogs were observed for 6 h after treatment for vomiting or other adverse reactions.

In accordance with the guidelines of WAAVP, [10, 25, 28], three fecal samples were examined by microscopic examination after zinc sulfate centrifugation to determine the presence of *Giardia* cysts 3 days before administering the drugs, as well as the day that treatments were administered (Day 0) and on days 5, 7, 9, 11, 14, 18, 25 & 28 [29]. The number of *Giardia* cysts per slide was quantified by microscopy and cyst scores (0–4) were determined as follows: 0 = 0 cysts per slide; 1 = 1–100 cysts per slide; 2 = 100–300 cysts per slide; 3 = 300–500 cysts per slide; 4 = TNTC (too numerous to count) per 10× objective field [30, 31]. Additionally, the flotation technique was conducted to detect *Cystoisospora* oocysts and nematode eggs. The Graham and Kinyoun techniques were carried out to detect *Dipylidium caninum* egg packets or *Cryptosporidium* oocysts, respectively [32, 33]. To exclude a subjective decision, each sample was analyzed by three trained technicians in coprological diagnosis.

Fecal consistency was determined on days 0, 5, 7, 9, 11, 14, 18, 25 and 28, according to the following scale: 1 = liquid diarrhea; 2 = doughy and shapeless; 3 = soft and well-formed; 4 = hard, dry, firm, smooth and well-formed; 5 = very hard and dry [34, 35]. To avoid a biased interpretation, three technicians that did not participate in this study scored each individual sample to obtain fecal consistency data.

### Biochemical parameters

Serum samples were processed to determine concentrations of aminotransferase alanine (ALT) and aspartate aminotransferase (AST), measured using a diagnostic kit (Spinreact, S.A. de C.V., Mexico) in a Cobas Mira analyzer (Roche, S.A. de C.V., Mexico), according to the manufacturer’s instructions.

### Statistical analysis

Results were adjusted with generalized linear models, and with maximum likelihood estimation methods [36]. Odds ratios were also estimated for the analysis of cyst and faecal categories for each treatment against the control group. An ordinal logit regression model was constructed to allow a better understanding of the study variables (cyst scores and faecal consistency).

## Results

Odds ratios (OR) calculated *vs* the control group are shown in Table 1. NTZ1, NTZ2, NTZ3 and FEB groups displayed diminished risk of shedding *Giardia* cysts as compared to untreated controls; yet NTZ2, NTZ3, and FEB were less likely to excrete *Giardia* cysts during the study (OR 0.559, 95% CI: 0.00–0.78, *P* = 0.001; OR 0.023, 95% CI: 0.015–0.036, *P* = 0.0001; OR 0.024, 95% CI: 0.015–0.038, *P* = 0.0001; OR 0.037, 95% CI: 0.024–0.038, *P* = 0.0001; respectively).

**Table 1:** Odds ratios (OR) and confidence intervals (CI) of cyst scores for groups treated with, or without, nitazoxanide or with pyrantel + praziquantel + febantel

Group	OR (*vs* CTRL)	95% CI	Times less than control (1/OR)	*P*
NTZ1	0.559	0.00–0.78	1.8	0.001
NTZ2	0.023	0.015–0.036	43.5	0.0001
NTZ3	0.024	0.015–0.038	41.6	0.0001
FEB	0.037	0.024–0.038	27.0	0.0001

*Abbreviations*: *NTZ1* group treated with 37.5 mg/kg nitazoxanide, *NTZ2* group treated with 75 mg/kg nitazoxanide, *NTZ3* group treated with 150 mg/kg nitazoxanide, *FEB* group treated with pyrantel + praziquantel + febantel, *CTRL* untreated group

No cysts were detected in the feces of groups NTZ2 and NTZ3 until day 11. On day 14, cysts were found in stool samples from one dog of each of these two groups. After the administration of NTZ to groups NTZ2 and NTZ3 on day 14, no cysts were observed until the end of the experiment. On the other hand, in the FEB group, one dog shed *Giardia* cysts on days 9, 11, and 14 and two dogs excreted no cysts on days 18, 25, and 28. Nevertheless, no significant difference was obtained between NTZ2, NTZ3 and FEB groups (*P* > 0.05). It was also found that the administration of NTZ or febantel did not result in different faecal consistencies in comparison to untreated controls.

Before the initiation of this trial, stool samples from all dogs were screened to identify infections with other parasites. Only *Giardia-*infected dogs were included in the study. However, stool samples from these dogs were positive for at least one of the following parasites: *Cystoisospora* spp., *Cryptosporidium* spp., *Toxocara canis*, *Ancylostoma caninum*, *Trichuris vulpis* or *Dipylidium caninum*. After an appropriate anthelmintic treatment, no cestodes or nematodes were detected in stool samples, while *Cystoisospora* oocysts were found in all groups, as no anticoccidial drug was administered to the experimental animals. As for the activity of NTZ against other parasites, dogs treated with 75 mg/kg or 150 mg/kg did not shed *Cryptosporidium* oocysts; whereas animals in NTZ1 or FEB groups remained infected with this protozoan group (Table 2).

**Table 2:** Coccidia and helminths of dogs detected in feces before and after treatment with nitazoxanide (NTZ) or a combination of pyrantel + praziquantel + febantel (FEB)

Group	Dog	Age (years)	Parasite	Day -3	Day 0	Day 14	Day 28
NTZ1	1	2	*Cystoisospora sp.*	+	+	+	+
			*Toxocara canis*	+	–	–	–
	2	1	*Ancylostoma caninum*	+	–	–	–
			*Toxocara canis*	+	–	–	–
	3	1	*Cryptosporidium*	+	+	+	+
			*Ancylostoma caninum*	+	–	–	–
	4	2	*Dipylidium caninum*	+	–	–	–
			*Toxocara canis*	+	–	–	–
			*Ancylostoma caninum*	+	–	–	–
	5	2	*Cystoisospora*	+	+	+	+
			*Dipylidium caninum*	+	–	–	–
			*Trichuris vulpis*	+	–	–	–
	6	2	*Ancylostoma caninum*	+	–	–	–
			*Trichuris vulpis*	+	–	–	–
	7	1	*Cystoisospora*	+	+	+	+
			*Cryptosporidium*	+	+	+	+
			*Toxocara canis*	+	–	–	–
			*Ancylostoma caninum*	+	–	–	–
NTZ2	8	1	*Cryptosporidium*	+	+	-	-
			*Ancylostoma caninum*	+	–	–	–
			*Trichuris vulpis*	+	–	–	–
	9	2	*Cystoisospora*	+	+	+	+
			*Toxocara canis*	+	–	–	–
			*Dipylidium caninum*	+	–	–	–
	10	1	*Ancylostoma caninum*	+	–	–	–
			*Trichuris vulpis*	+	–	–	–
	11	1	*Cystoisospora*	+	+	+	+
			*Ancylostoma caninum*	+	–	–	–
			*Toxocara canis*	+	–	–	–
	12	2	*Cystoisospora*	+	+	+	+
			*Trichuris vulpis*	+	–	–	-
			*Dipylidium caninum*	+	–	–	–
	13	1	*Cryptosporidium*	+	+	-	-
			*Cystoisospora*	+	–	+	+
			*Ancylostoma caninum*	+	–	–	–
			*Dipylidium caninum*	+	–	–	–
	14	1	*Ancylostoma caninum*	+	–	–	–
			*Toxocara canis*	+	–	–	–
			*Trichuris vulpis*	+	–	–	–
NTZ3	15	1	*Cystoisospora*	+	+	+	+
	16	1	*Cryptosporidium*	+	+	-	-
			*Cystoisospora*	+	+	+	+
			*Toxocara canis*	+	–	–	–
	17	1	*Ancylostoma caninum*	+	–	–	–
			*Toxocara canis*	+	–	–	–
			*Trichuris vulpis*	+	–	–	–
			*Dipylidium caninum*	+	–	–	–
	18	1	*Ancylostoma caninum*	+	–	–	–
	19	2	*Cryptosporidium*	+	+	-	-
			*Cystoisospora*	+	+	+	+
			*Trichuris vulpis*	+	–	–	–
	20	2	*Ancylostoma caninum*	+	–	–	–
			*Trichuris vulpis*	+	–	–	–
	21	1	*Cryptosporidium*	+	+	-	-
			*Trichuris vulpis*	+	–	–	–
			*Dipylidium caninum*	+	–	–	–
FEB	22	2	*Cryptosporidium*	+	+	+	+
			*Dipylidium caninum*	+	–	–	–
	23	1	*Cystoisospora*	+	+	+	+
			*Toxocara canis*	+	–	–	–
	24	1	*Cystoisospora*	+	+	+	+
			*Ancylostoma caninum*	+	–	–	–
			*Trichuris vulpis*	+	–	–	–
	25	1	*Cryptosporidium*	+	+	+	+
			*Ancylostoma caninum*	+	–	–	–
	26	1	*Cryptosporidium*	+	+	+	+
			*Ancylostoma caninum*	+	–	–	–
			*Trichuris vulpis*	+	–	–	–
	27	1	*Cystoisospora*	+	+	+	+
			*Toxocara canis*	+	–	–	–
			*Ancylostoma caninum*	+	–	–	–
	28	1	*Toxocara canis*	+	–	–	–
CTRL	29	2	*Dipylidium caninum*	+	–	–	–
			*Toxocara canis*	+	–	–	–
			*Ancylostoma caninum*	+	–	–	–
	30	2	*Cryptosporidium*	+	+	+	+
			*Cystoisospora*	+	+	+	+
			*Dipylidium caninum*	+	–	–	–
			*Trichuris vulpis*	+	–	–	–
	31	1	*Toxocara canis*	+	–	–	–
			*Ancylostoma caninum*	+	–	–	–
	32	1	*Cystoisospora*	+	+	+	+
			*Toxocara canis*	+	–	–	–
			*Ancylostoma caninum*	+	–	–	–
	33	2	*Cryptosporidium*	+	+	+	+
			*Toxocara canis*	+	–	–	–
	34	2	*Toxocara canis*	+	–	–	–
	35	1	*Cystoisospora*	+	+	+	+
			*Dipylidium caninum*	+	–	–	–
			*Toxocara canis*	+	–	–	–

*Abbreviations*: *NTZ1* group treated with 37.5 mg/kg nitazoxanide, *NTZ2* group treated with 75 mg/kg nitazoxanide, *NTZ3* group treated with 150 mg/kg nitazoxanide, *FEB* group treated with pyrantel + praziquantel + febantel, *CTRL* untreated group

The therapeutic protocols used in this study did not affect AST and ALT levels in any dog from NTZ1, NTZ2, NTZ3, and FEB groups. Regarding the overall health of experimental animals, vomiting occurred in a single dog treated with 150 mg/kg nitazoxanide during the treatment administration on day 14.

## Discussion

Options for the treatment of giardiasis in dogs are limited, and the few available drugs are often accompanied by adverse reactions [12]. For example, it has been documented that metronidazole administered to dogs at a rate of 22 mg/kg, twice daily for 5 days, induces vomiting and neurological dysfunctions. Additionally, this drug has mutagenic and carcinogenic potential [1, 37]. Albendazole has been linked to vomiting, bloody diarrhea, abortion, and teratogenicity [38]. Thus, the only available option at present for the treatment of canine giardiasis is febantel; a drug usually combined with praziquantel and pyrantel, administered for three consecutive days, as in the FEB group in this trial.

This study indicates that the anti-*Giardia* activity of 75 mg/kg or 150 mg/kg NTZ or the combination of antiparasitic drugs containing febantel, was similar. This finding is consistent with previous reports that document the giardicidal efficacy of febantel [28, 38, 39]. Nevertheless, results obtained in the present trial demonstrate that cysts were recovered from the FEB-treated dogs six days after the last treatment. This observation agrees with previous studies that showed an increase in the number of dogs shedding cysts and the number of cysts shed by dogs, beginning six days after treatment with this same combination [38]. Other reports state that there appears to be no significant improvement when febantel is given to *Giardia*-infected dogs for three or five consecutive days [28]. On the other hand, the administration of 75 mg/kg or 150 mg/kg NTZ in the present study, halted the shedding of *Giardia* cysts by dogs for 13 days. Hence, it is reasonable to suggest that a single dose of NTZ at 75 mg/kg or 150 mg/kg results in reduced shedding of cysts when compared to febantel. In contrast, the data generated in the present study shows a lack of a statistically significant difference between cyst output in dogs that received a single dose of NTZ at 37.5 mg/kg when compared to untreated controls.

To the best of our knowledge, this is the first study to confirm the high efficacy of NTZ for reducing *Giardia* cyst shedding in dogs. A previous study documented shedding of *Giardia* cysts in infected dogs five days after the administration of NTZ at a dosage of 25 mg/kg two times a day*.* [26]. In the present study, the cyst shedding pattern of the NTZ1 (37.5 mg/kg) group was similar to that of the untreated controls during the experiment. In contrast, dogs that received a single dose of 75 mg/kg or 150 mg/kg, showed a reduction in cyst shedding. In this case, the presence of parasite cysts after treating dogs could be attributable to reinfection. *Giardia* has a short prepatent period of 5 days and cysts are resistant to disinfectants and environmental conditions. These factors, together with stress and the presence of cysts in the animal’s fur, may explain reinfection [38].

Regarding the effect of the administration of NTZ or febantel on faecal consistency, the results of this study do not concur with previous findings, which demonstrated that faeces could be shapeless to diarrheic in untreated dogs as compared to febantel treated dogs [28]. However, all *Giardia*-infected dogs were also infected with at least one helminth or protozoan, such as *Cystoisospora* spp. which did not respond to NTZ treatment. Infections caused by coccidia are associated with abnormal faecal consistency and deterioration of faecal scores [40, 41]. The presence of coccidia in the stool of dogs could account for the abnormal consistency of stool specimens despite anti-*Giardia* treatment, supporting the hypothesis of the potential significance of cystoisosporosis as an associated cause of diarrhea in dogs. One major limitation of this study was the lack of an uninfected control group, which could have confirmed the absence of a clear association between fecal consistency and *Giardia* infection [42–45].

In addition to the observed giardicidal activity of nitazoxanide administered at 75 mg/kg or 150 mg/kg, anti-*Cryptosporidium* activity was observed. This finding coincides with data reported in previous experiments, which demonstrated that nitazoxanide dosages ranging from 50 to 250 mg/kg reduce *Cryptosporidium* oocyst excretion in laboratory animals and goats [46]. It should be noted that nitazoxanide is the only drug approved by the FDA in humans, as a treatment option for cryptosporidiosis [47].

Dogs kept in kennels or shelters are more susceptible to suffer from anorexia, anemia, and diarrhea caused by gastrointestinal helminths and protozoans, given that these places include young, old, and immunocompromised animals. Indeed, co-infection with several intestinal parasites is frequently reported in kennels and shelters, involving parasites such as *Giardia, Toxocara canis, Cryptosporidium* spp.*, Ancylostoma caninum, Capillaria aerophila* [48], *Toxascaris leonina*, *Uncinaria stenocephala, Trichuris vulpis, Sarcocystis* spp., capillariid eggs [49, 50], and *Cystoisospora* spp. [51]. Therefore, broad-spectrum drug formulations are most suitable in these cases [41]. Indeed, NTZ administered at a dose of 75 mg/kg could represent a suitable drug for use under these circumstances.

In this work, virtually no adverse reactions were observed, much in agreement with other studies [23]. Vomiting occurred in one dog after the administration of 150 mg/kg of NTZ. This result agrees with the study by Basu et al. [52], who reported nausea in 9% of patients treated with this compound. This may suggest that the lower dose of 75 mg/kg is marginally safer, though further research is required. In contrast, it has been proposed that fenbendazole, the active form of febantel, may promote liver tumors because it induces upregulation of cytochrome P-450 isozymes (CYP) 2B1/2, which are involved in hepatocarcinogenesis [53]. Finally, it is important to reiterate that the therapeutic use of antiparasitic drugs in kennels and shelters, should not be the only measure employed to control these infections. Dog bathing and the frequent cleaning and disinfection of their environment, reduces the risk of infection and re-infection [28]. In fact, studies have demonstrated that dogs re-shed cysts following a brief period after antiparasitic treatment when no disinfection or cleaning of their enclosures is performed. Shampooing of dogs is recommended because fecal material on the fur increases the chance of reinfection [54–57].

The results of the current study suggest that administration of 75 mg/kg of NTZ every 14 days is effective for the treatment of giardiasis and cryptosporidiosis in dogs, though this requires further evaluation.

## Conclusions

This study demonstrates that the administration of 75 mg/kg of NTZ every 14 days to dogs infected with *Giardia* reduces cyst excretion. Data from this study will help in the development of *Giardia* control programmes, particularly in shelter animals.

## Abbreviations

CTRL: Non-treated group
FEB: Group treated with pirantel + praziquantel + febantel
TNTC: Too numerous to count.
NTZ: Nitazoxanide
NTZ1: Group treated with 37.5 mg/kg nitazoxanide
NTZ2: Group treated with 75 mg/kg nitazoxanide
NTZ3: Group treated with 150 mg/kg nitazoxanide
